# Odontogenic Myxoma of Maxilla in an Atypical Location: A Case Report

**Published:** 2013-03

**Authors:** P Ghalayani, GR Jahanshahi, HR Mohagheghiyan

**Affiliations:** a Dept. of Oral Medicine, School of Dentistry, Isfahan University of Medical Science, Isfahan, Iran; b Dept. of Pathology, School of Dentistry, Isfahan University of Medical Science, Isfahan, Iran; c Young Researchers Club, School of Dentistry, I.A.U, Khorasgan Branch, Isfahan, Iran

**Keywords:** Odontogenic Myxoma, Benign Odontogenic Tumor, Myxoma

## Abstract

Odontogenic myxoma is a rare and locally invasive benign neoplasm found exclusively in jaws. It presents local invasiveness and tendency to recurrence. According to the World Health Organization (WHO), the odontogenic myxoma is classified as an odontogenic tumor of ectomesenchymal origin. The odontogenic myxoma is a rare entity found in both jaws while the mandible is involved more commonly than the maxilla. We present a kind of odontogenic myxoma in a 24-year old male that was found in an unusual location.

## Introduction

Odontogenic myxomas are tumors derived from embryonic mesenchymal elements of dental anlage. It appears to originate from the dental papilla, follicle, or periodontal ligament [1]. The evidence for its odontogenic origin arises from its almost exclusive location in the tooth- bearing areas of the jaws, its occasional association with missing or unerupted teeth and the presence of odontogenic epithelium [2].

Odontogenic myxoma is a locally invasive lesion that does not metastasize and displays slow and asymptomatic growth, sometimes resulting in expansion or even perforation of the cortex of the involved bone [3]. It is composed of round and spindle-shaped cell lying in an rich myxoid stroma [4]. The tumor is rich in extracellular matrix [ECM] represented by type I collagen, fibronectin, tenascin, chondroitin sulfate and especially rich in hyaluronic acid [5-7]. Small islands of apparently inactive odontogenic epithelial rests may be scattered through the myxoid substance [8-9]. There is a microscopic similarity between odontogenic myxoma and the dental papilla [10]. According to Kim j et al [11] myxomas do not have the epithelial lining found in many dental follicles. Odontogenic Myxoma constitutes about 3-6% of total odontogenic tumors. Odontogenic Myxoma occurs commonly in the mandible and their presentation in the maxilla is rare [12]. Radiographically, the tumor presents as a unilocular or multilocular radiolucent lesion with well-defined borders and fine, bony tra-beculae within its interior structure, expressing as “honeycombed”, “soap bubble", or “tennis racket” appeara-nce. Unilocular appearance may be seen more commonly in children and in the anterior part of the jaws [2, 13-14]. Dental displacement is a relatively common finding.

Recommended therapy varies from curettage to radical excision, complete surgical removal can be difficult as the lesion is not encapsulated and because the myxoamatous tissue infiltrates adjacent bone tissue. These characteristics may explain the high rate of recurrence of myxomas, which ranges from 10 to 33% with an average of 25% [3, 13].

## Case report

A 24-year old male referred to the department of oral

**Figure 1:**
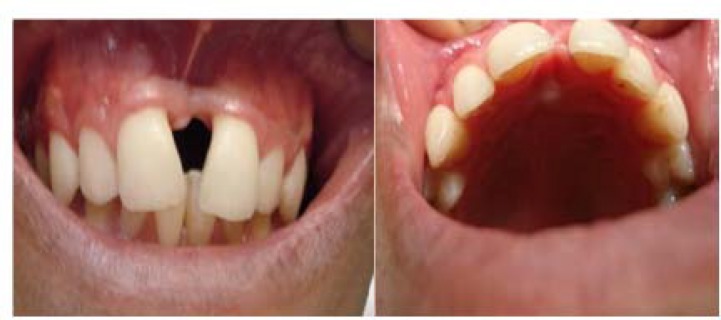
Intraoral examination revealing no buccal and palatal swelling

**Figure 2:**
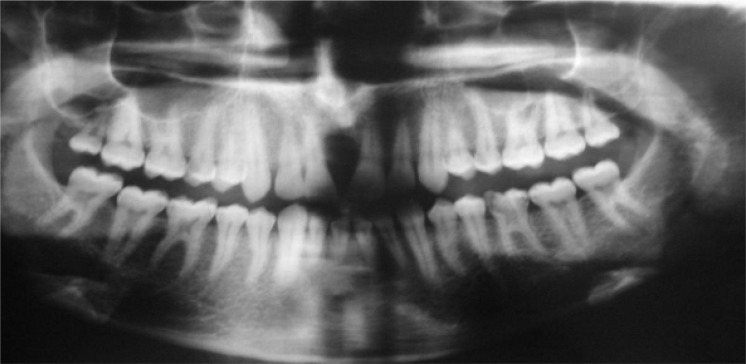
Panoramic radiograph showing a unilocular radiolucent area between central incisors. Note the roots of two central incisors are displaced distally

medicine, Esfahan Dental School (Esfahan University of Medical Sciences) for orthodontic treatment, complaining of diastema between his anterior teeth. Initially, the patient had no signs and symptoms such as pain and swelling (Figure 1).

**Figure 3:**
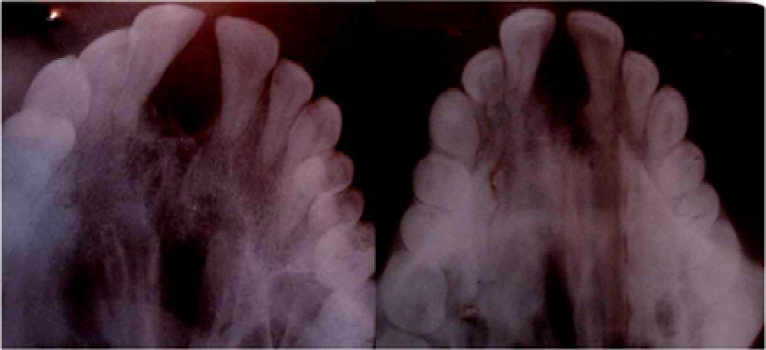
Occlusal radiograph revealed the unilocular appearance of the lesion

The Panoramic radiography showed resorption of the bone in the region of the incisor teeth (Figure 2). However occlusal radiography revealed a well-demarca-ted unilocular radiolucent lesion with pear-shaped appearance involving the incisive canal region (Figure 3). 

Radiographic differential diagnoses were incisive canal cyst, odontogenic keratocyst, lateral periodontal cyst, nasopalatine cyst and central giant cell granuloma. Extraoral examination showed no significant swelling in the anterior portion of the maxilla. The mucosa over the lesion was normal and there was no hotness in the region. The vitality test has been performed and revealed the teeth were vital. There was not any swelling, and the patient had no history of paresthesia.

In an open surgery, it has been found a gelatin solid lesion with well-defined extension in incisive canal region, without invading the bone.

**Figure 4a:**
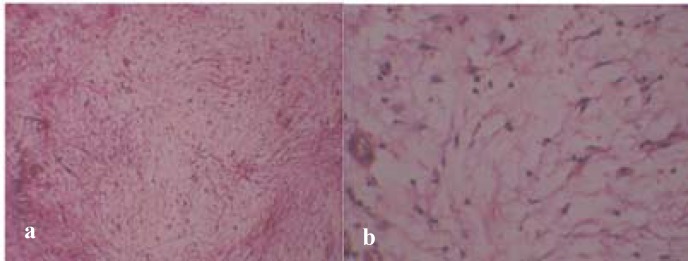
Myxomatous tissue showing fine collagen fibers **b **Collagen fibers and spindle polyhedral cells dispersed in myxomatous tissue

Histopathological examination of the excised specimen showed a tumor mass without encapsulation. It showed round-and spindle-shaped cells in loose, abundantly myxoid connective tissue stroma, closely resembling the mesenchymal portion of a developing tooth. Some areas showed a moderately dense of fibrous tissue (Figure 4a and b).

**Figure 5a:**
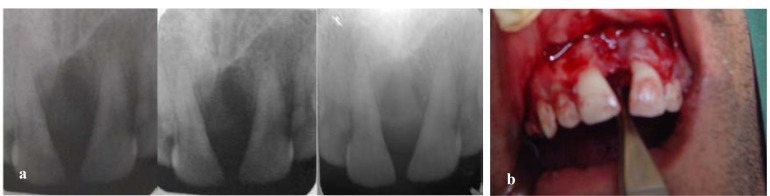
Periapical radiograph after six month follow- up. There were no findings of recurrence **b **There was no sign of recurrent after six months follow-up

An excisional biopsy has been performed and the tumor removed completely. After surgery the patient, has been followed by periodic radiological examinations, together with intraoral and clinical examination in three- month intervals (Figure 5a) and there was no sign of recurrence (Figure 5b).

## Discussion

Odontogenic myxoma (OM) is a non-capsulated benign tumor of the jaws that rarely occurs. It is derived from the dental mesenchyme or periodontal ligament. Previous studies showed the highest incidence in the third decade of the life and the majority of the cases were between 10 and 40 years old [15]. This lesion may be infiltrative, aggressive, and maybe recurred [2, 16].

OM almost exclusively occurs in the jawbones, comprising around 3-6% of all odontogenic tumors. The tumors occur in a range of ages that varies from 22.7 to 36.9 years, compatible with our patient’s age. It is rarely seen in patients younger than 10 years old or older than 50 [17]. Its frequent location is the posterior mandible, however, other locations such as the incisive region, maxilla and mandibular condyle must be considered [1], and in this case we find the lesion in the anterior portion of the maxilla, in the incisive canal region. Our study depicts a rare example of OM in this area as well.

The mandible appears to be more frequently involved than the maxilla, especially in the posterior region; in this case, the anterior region of the maxilla is implicated.

Myxomas are almost always asymptomatic, although some patients present progressive lesions involving maxilla and maxillary sinus, with eventual neurological disturbance. The presence of pain, paresthesia, ulceration and dental mobility has been noted in the literature [18-19].

Odontogenic myxomas can be classified into two types, central myxoma located in the bone or peripheral myxoma located extra-osseous or in the soft tissue overlying the tooth-bearing areas [20].

A review of the literature between 1965 and 1995 by Kaffe et al [22], disclosed a total number of 164 cases of odontogenic myxomas with relevant information about the age, gender, and location. The tumors occur more often in females (100 cases [61%]) than in males (64 cases [39%]) and are located in the mandible in two thirds (109 cases) and in the maxilla in one-third (55 cases) of cases. Despite the differences in reported location, most odontogenic myxomas occur in 

the premolar and molar region of both jaws.

In the other study by Tie-Jun et al [23], posterior tumors in both the maxilla and mandible tended to be larger and more destructive with frequent involvement of the maxillary sinus (9 of 10 cases) and the mandible ramus (6 of 9 cases). Twenty-three of 25 tumors occupied only one side of the jaws (right or left). This concurs with the notion that odontogenic myxoma rarely crosses the midline [22, 24].

Raubenheimer et al reported two cases of peripheral odontogenic myxoma gingival soft tissue without involving the bone. . This study showed that they form a distinctive, even though rare, clinical entity with a potential to grow into large disfiguring lesions [25].

A study by Saghravanian et al showed that among 8,766 patients, only 165 odontogenic tumors (1.9%) were found, with a mean age of 26.3 years (range 6-81 years). One hundred and fifty-eight tumors were central with high incidence in the posterior region of both jaws especially in the mandible and seven were peripheral tumors, including five in the posterior mandible and two in the anterior maxilla. Although the prevalence of odontogenic tumors varies in different geographic sites, there are few reports on the relative frequency of odontogenic tumors in Iran [21].

In this presentation odontogenic myxoma occur in the 24-year old male, in the anterior portion of the maxilla and it is different with other tumors which were reviewed in the previous studies the lesion presented as a rare odontogenic myxoma, especially the particular site of this tumor. Since a radiolucent region with well-defined unilocular shape was seen in the incisive canal area, the first diagnosis was an incisive canal cyst; while we considered other diagnoses as well.

Despite of being a benign tumor, the odontogenic myxoma presents a high recurrence rate of about 25% [26]. Suarez PA et al related this phenomenon to the encapsulation of the lesion or to the ability of tumor cells to penetrate through bone trabeculae [27].

Radiographically, OMs appear as multilocular or unilocular radiolucencies. Unilocular lesions are more frequently found in the anterior region of the jaws, while multilocular lesion occurs mainly in the posterior region [17]. Unilocular lesions were mostly located in the anterior and multilocular in the posterior areas of the jaws. Because the radiological appearance of myxoma may be essentially similar to many other lesions of the jaws; thorough radiological examination is compulsory for planning a proper treatment [28].

Odontogenic myxoma should be included in the differential diagnosis of both radiolucent and mixed lesions, in both jaws, and for all age groups.

When unilocular and without the trabeculae, the tumor closely resembles periapical , lateral periodontal and traumatic bone cyst, when multilocular , it must be distinguished from ameloblastoma, central heamagioma and odontogenic keratocyst and central giant cell granuloma [29].

Clinical and radiological aspects of odontogenic myxomas are not conclusive; a histopathological examination of the lesion is compulsory to make the right diagnosis [30].Finally, odontogenic myxoma has a variable clinical and radiological appearance and it should be considered in the differential diagnosis of radiolucent and mixed radiolucent-radiopaque lesions of both jaws in all age groups [22].
